# “I want to be there. I have to be there.”: Parents’ perceived barriers and facilitators to bedside presence in the pediatric intensive care unit

**DOI:** 10.3389/fped.2023.1308682

**Published:** 2024-01-08

**Authors:** Emily I. Poole, Molly Ryan, Martha Walls, Corey Slumkoski, Janet A. Curran, Jamie A. Seabrook, Jennifer R. Foster

**Affiliations:** ^1^ Faculty of Medicine, Dalhousie University, Halifax, NS, Canada; ^2^ Department of Critical Care, Dalhousie University, Halifax, NS, Canada; ^3^ Pediatric Critical Care Patient Partnerships, IWK Health, Halifax, NS, Canada; ^4^ School of Nursing, Dalhousie University, Halifax, NS, Canada; ^5^ Department of Pediatrics, IWK Health, Halifax, NS, Canada; ^6^ Department of Pediatrics, Western University, London, ON, Canada; ^7^ Department of Epidemiology and Biostatistics, Western University, London, ON, Canada; ^8^ School of Food and Nutritional Sciences, Brescia University College at Western University, London, ON, Canada; ^9^ Department of Pediatric Critical Care, IWK Health, Halifax, NS, Canada

**Keywords:** critical care, pediatric, parental presence, family-centred care, patient visitors, intensive care unit, qualitative

## Abstract

**Introduction:**

Parental presence at the bedside during a stressful pediatric intensive care unit (PICU) admission may improve child comfort, reduce parental anxiety, and enable family engagement. We performed this study to identify factors that parents perceive impact their capability, opportunity, and motivation to be at the bedside in PICU.

**Methods:**

We conducted a qualitative descriptive study using semi-structured interviews based on the Theoretical Domains Framework (TDF). We included parents of children admitted to the PICU for at least 24 h at IWK Health in Nova Scotia, Canada. Interviews were coded independently by two researchers using a directed content approach based on the TDF. We generated themes and subthemes, with the subthemes identified as factors impacting parental presence, and assigned TDF domains to each of the subthemes.

**Results:**

Fourteen primary caregivers (8 mother figures, 6 father figures) participated in 11 interviews. The factors associated with parental presence were captured by 6 themes: *Understanding the Medicalized Child*; *Maintaining the Parent Role; Life Beyond the Hospital; Parental Intrinsic Responses and Coping; Support Structures;* and *The PICU Environment*. Fifty-two barriers and enablers were identified within 13 TDF domains; 10 TDF domains were determined to be relevant to parental presence, which may be used to guide design of future interventions. Participants emphasized the importance of self-care to enable them to remain physically at their child's bedside and to be engaged in their care.

**Conclusions:**

Parents perceive multiple factors within 6 themes act as barriers or enablers to presence with their critically ill child in the PICU. Guided by relevant TDF domains, interventions may be designed to optimize presence, particularly engaged presence, which may improve health-related outcomes of children and their parents.

## 1 Introduction

Admission to the pediatric intensive care unit (PICU) is frightening for both parent and child. It has frequently been described as other-worldly, or a journey into the unknown (1, 2). Although critical care medicine is often invasive and can be overwhelming for families, parental presence has been linked to both reduced parental anxiety (3) and improved child comfort (4), and is necessary for operationalizing a family-centred care model (5, 6).

Parents want to be with their critically ill child. Parents have cited separation from their child and parental role alteration as significant stresses during a PICU stay (7, 8). Parents commonly identify being close to their child as a primary parental need and something that improves their overall PICU experience (1, 9, 10). One study demonstrated that mothers who were allowed individualized (flexible and lengthy) presence with their child had significantly lower anxiety than those who were required to follow strict visitation hours (3). Children and youth express decreased psychologic stress when their parents are present (4) and may have increased comfort when their parents participate in their care (11).

Most research on parental presence in the PICU has focused on specific events like rounds or painful procedures (12, 13), rather than routine PICU care. A single-centre study in a Canadian PICU with unrestricted visitation found that children had a parent present approximately 60% of the time, with younger and sicker children being less likely to have a parent present than those who were older and more stable (14). Although that study provided correlations between time at the bedside and factors extrinsic to the parent, the factors that impact motivation, capability, and opportunity to be at the bedside remain unclear.

Several studies have explicitly examined factors affecting parental presence in the neonatal intensive care unit (NICU). Heinemann et al. found that barriers to parental presence included the NICU's physical environment and painful procedures being performed (15). The main barriers identified by Wigert et al. were ill health of the parents, a non-family-friendly environment, and practical factors such as the family's financial situation or obligations at home (16). Parental participation in care (e.g., through kangaroo mother care), coupled with easy access to the NICU via convenient accommodations and a family-friendly environment facilitated presence (15, 16). While there are likely similarities between family member experiences in the NICU and PICU, the context and culture in the units are quite different and parental experiences may differ between a newborn and a child who is already incorporated into family life.

Pediatric-specific research is necessary to explore parental perceptions of barriers and facilitators of presence with their critically ill child in the PICU with the goal of identifying areas for potential interventions. While there are multiple factors external to the parent that may reasonably be expected to impact presence (e.g., policy and PICU room design), we hypothesized that there are also multiple psychosocial factors that could only be explored by asking participants about their experiences. Using social and behavioral theories to guide exploration may help identify determinants amenable to intervention. Therefore, we chose to explore parents’ own perceptions of barriers and enablers to their presence in the PICU through interviews guided by the Theoretical Domains Framework (TDF;17). The TDF is a comprehensive framework designed to identify determinants of behavior within 14 domains, and expands on the determinants of behavior change identified by the COM-B (Capability, Opportunity, Motivation) model of behavior change (17). The framework may be used to identify individual and system-level factors amenable to intervention and change (18, 19).

## 2 Methods

### 2.1 Design

We performed a descriptive, qualitative study that was informed by the TDF to explore parental perceptions of the factors that may be barriers or enablers to their presence at the bedside of their critically ill child in the PICU. We followed the consolidated criteria for reporting qualitative research (COREQ) to structure our study report, presented in Supplementary Material File S1 (20). This study was approved by the IWK Research Ethics Board (#1025911).

### 2.2 Study team reflexivity

Characteristics of the study team such as occupation, gender, and training are outlined in a reflexivity Supplementary Material File S2. EP, JF, and MR are cis-gendered white females who live and work on unceded Mi'kmaw territory in the Atlantic region of Canada. The research team reflected that their approach to participants and interpretations may have been rooted in a colonial bias to focus on positive social stereotypes of white participants and provide negative interpretations with respect to the opportunities, capabilities, and motivations of non-white or socio-economically disadvantaged participants. The researchers reflected that their gender may have biased both the way in which questions were asked of mothers compared to fathers and their interpretations of traditionally gender-based roles and expectations.

### 2.3 Population and setting

We included parents of pediatric patients (birth to 16 years) who were admitted to the IWK Health PICU for at least 24 h. “Parent” was defined by self-identification as the primary parent(s) regardless of sex, gender, or family composition. Up to two primary parents were included per child. We purposively sampled for type of admission (planned vs. unplanned), presence of complex chronic illness, patient sex, parent gender, and the patient's age group [infant (less than 1 year), child (1–12 years), and teen (13–16 years)]. Participants were excluded if they were not fluent in English, if their child was admitted based on suspected child abuse, or if their child was expected to die during the admission. Recruitment and data analysis were carried out in parallel and iteratively. We sampled until we had an adequate sampling frame and had reached data saturation, with no new themes generated for two interviews.

Participants were not known to EP prior to the study. Participants first provided a care team member consent to be approached by a researcher, and then discussed the study in person with EP, who explained the study purpose and procedures and provided a written letter of information. All participants provided their voluntary, informed, written consent prior to participation.

The PICU at IWK Health is a 10-bed, single-room care unit that provides mixed cardiac and medical-surgical services. Parents are provided sleeping accommodations both within each patient room and in separate parent sleeping rooms. Parents have unrestricted ability to come and go from the patient room, the PICU, and the hospital and are provided with a swipe card to access the PICU.

We screened for and enrolled participants between March 1, 2021 and April 30, 2022, during the COVID-19 pandemic. Although we had planned to start enrolling on May 1, 2020, we paused initiation until policy had evolved to enable both parents simultaneous, unlimited access to their child. All patients were limited to two support people at a time. Support people could trade with another family-identified adult once a week for the first half of the study, and at will for the rest of the study. Support people's mobility within the hospital and ability to leave the hospital was unrestricted. Non-adult siblings were prohibited.

### 2.4 Data collection

We designed an interview guide based on the 14 domains of the TDF (18). Adjustments were made to the interview guide at the start of enrollment to acknowledge and address the impacts of restrictions to family presence implemented at all Canadian pediatric hospitals during the COVID-19 pandemic. We added questions to the interview guide as the analysis progressed to improve trustworthiness by exploring experiences and descriptions with subsequent participants. See Supplementary Material File S3 for the interview guide including added questions. Participants also completed a demographic questionnaire and the Medical Term Recognition Test (METER). The METER is a brief, self-administered assessment of health literacy with scores categorized as functional (scores 35–40), marginal (scores 21–34) and low health literacy (scores 0–20) as per Rawson et al. (21).

The Principal Investigator (EP) received coaching on conducting interviews by JF and JC prior to the first interview and performed all interviews. EP and JF met every 2–3 interviews to debrief. Interviews were conducted when participants’ children were nearing the end of their PICU stay and took place in either a private conference room or at the child's bedside, at participants’ preference. Participants first completed the METER and demographic form and then participated in a 30–60 min interview which was audio recorded. EP took field notes of each encounter immediately after the interview to document non-verbal communication and as part of reflexivity practice. Though field notes informed interpretation of some quotes, they were not formally included in the coding and analysis. Participants’ transcripts were transcribed verbatim, then returned to participants for the opportunity to review the transcript, correct errors, and remove or elaborate on statements. No study participants opted to make changes to their interview transcript. Participants’ contributions were compensated with a gift card.

### 2.5 Analysis

Categories for demographic questionnaire responses and METER scores were created *a priori*. Demographic data are presented descriptively using median (range) as appropriate.

Coding followed a directed content analysis approach (22) with TDF domains as the coding framework. Verbatim transcripts were anonymized for identifying data and imported into NVivo 12 (QSR International). EP and JF initially read the interview transcripts several times to immerse in the data, then highlighted text that was related to parental presence and coded the data using the TDF domains. Analysis proceeded concurrent with interviews. EP and JF met after the first 2 interviews to discuss coding approaches, develop the codebook, confirm the fit of the TDF framework and to discuss potential themes that were not addressed in the TDF, and then met in an iterative process after every 3–4 interviews. EP kept memos of each coding meeting, including the relevant discussion, codebook updates, and emerging themes and the team maintained a reflexivity document.

After initial deductive coding of quotes using the TDF, EP and MR then inductively coded the quotes within each domain into a set of factors that represented potential barriers and enablers to parental presence in the PICU. These were reviewed and refined by JF. Potential factors and associated quotations were reviewed by parent partners MW and CS, and their feedback was incorporated into the analysis. EP then grouped the potential factors into themes and sub-themes of barriers and enablers to parental presence and created descriptions for each; these were reviewed, revised, and ultimately agreed upon by JF and MR with differences being resolved through discussion. EP and JF selected quotes to highlight each theme and subtheme and refined the theme and subtheme descriptions through manuscript preparation.

Assignment of relevant TDF domains for each sub-theme was done by linking the quotes associated with each subtheme with the original deductive coding scheme. As part of the directed content analysis, JF and EP then reassessed the fit of each assigned TDF domain for each given theme and sub-theme. Consistent with other authors, a TDF domain was considered relevant and a potential target for intervention if: (1) Multiple participants identified the factors associated with the domain as relevant; (2) There were conflicting participant statements within a theme/sub-theme; and (3) The themes and sub-themes within a domain had a strong influence on parental presence at the PICU bedside, based on participant statements (23).

## 3 Results

We conducted 11 interviews with 14 parents (8 self-identified mothers, 6 self-identified fathers) whose children were admitted to the PICU between March 4, 2021, and April 30, 2022. Three sets of parents chose to complete the interview together but were considered individual participants (throughout the manuscript these participants are identified as M = mother, F = father). The length of admission at the time of interview ranged from 3 to 15 days. The median parental METER score was 36.5 (range 4–40) and ranges are demonstrated with demographic data in Table 1.

**Table 1:** Participant and child demographics.

Demographic category	Number of respondents
Admission type (*n* = 11)	
Planned	4
Unplanned	7
Child sex (*n* = 11)	
Male	7
Female	4
Child age (*n* = 11)	
Infant (<2 years)	6
Child (2–12 years)	2
Teenager (>12 years)	3
Previous PICU admission (*n* = 11)	
Yes	4
No	7
Presence of chronic health condition (*n* = 11)	
Yes	7
No	4
Goals of care discussion during admission (*n* = 11)	
Yes	6
No	5
Distance from home to PICU (*n* = 11)	
100 km or less	4
>100 km	7
Parent age (years; *n* = 14)	
<25	3
25–50	9
>50	2
Self-identified parent role (*n* = 14)	
Mother	8
Father	6
Presence of other children at home (*n* = 14)	
Yes	10
No	4
Parent relationship status (*n* = 14)	
In a relationship with other primary parent	12
Not in a relationship with other primary parent	2
METER scores	
Functional health literacy	8
Marginal health literacy	4
Low health literacy	2

We identified six themes, which included 53 barriers and enablers that were relevant to parental presence at the bedside of critically ill children in the PICU. These are demonstrated along with a representative quote for each theme in Table 2. Representative quotes for each subtheme, or factor identified as a barrier and enabler, are provided in Supplementary Material File S4. We did not define the nature of each factor as a barrier or an enabler, as a given factor may act as either for a given individual. We assigned each subtheme to one or more relevant TDF domains; there were 98 domain assignments (Table 2). The most represented domain was *Environmental Context and Resources* (*n* = 19) which was represented in five themes. This was followed by *Social Influences* (*n* = 15) which was represented in at least one subtheme within all six themes and by all of the subthemes in the *Support Structures* theme. Ten domains were found to be relevant for potential interventions to address and optimize parental presence at the bedside. Four domains—*Behavioral Regulation*, *Skills*, *Optimism,* and *Intentions* did not meet the threshold for relevance.

**Table 2:** Themes and subthemes with descriptions and TDF domain assignments.

**Theme: The Medicalized Child** **Description:** Parents’ knowledge and understanding of their child and the child's medical status including the child's interactions with the PICU, which may impact the capability, motivation, and opportunities for presence. “It's just a lot of tubes and monitors, and people coming in and out, and beeping, and all the things hooked up to her, that's like the scariest part because she's so tiny. And just seeing like all the wires and hoses. She doesn't have much on her now but yesterday she had a lot more. And it's just hard to see her kind of hooked up to that. And she was sedated for a bit. So for her, just to see her just kind of not really responding to us, and that kind of thing was really hard.” (Participant 8)		
**Subthemes: Factors identified as barriers or enablers to parental presence**		**TDF Domain**
Information needs	**Medical or PICU experience and knowledge**: Previously held experiences and knowledge that impact the ability, motivation and opportunity for presence	Knowledge Beliefs about capabilities
	**Preparation for appearance of critically ill child in PICU**: Whether, how, and degree to which the parent was prepared for seeing their critically ill child in the PICU context	Knowledge
	**Opportunities to gain information**: How presence affects the parent gaining information about the child or PICU	Reinforcement
Child's clinical course	**Knowledge of the child's baseline and disease process**: Knowledge parents have of their child and their child's health status	Knowledge Beliefs about capabilities
	**Preparation for child's clinical course and outcome**: Prior information provided to prepare parents for the clinical course and PICU stay and to keep them abreast of the child's prognosis	Knowledge
	**Child's medical status**: Degree, type, stage, and details of the child's medical status	Environmental context and resources Emotion
	**Hope for recovery**: Optimistic or pessimistic thinking about the child's clinical course and outcome	Optimism
Witnessing the child's response to PICU	**Child's responses to care**: The child's behaviors, emotions, and interactions that may impact presence	Emotion Environmental context and resources
	**Child's expectations**: Expectations children have of their parents’ presence or absence in PICU	Social influences
**Theme: PICU Parent Role** **Description:** The parenting identities, roles, and responsibilities that parents perceive while their child is in PICU as related to the parent's motivation, opportunities, and capability to be present at the bedside “I'm just doing what I'm supposed to be doing as a father—taking care of my child.” (Participant 9)		
**Subthemes: Factors identified as barriers or enablers to parental presence**		**TDF Domain**
Perceived roles and responsibilities while their child is in PICU	**Advocating for the child:** Advocating with the medical team in the child's perceived best interest	Beliefs about capabilities Social/professional identity and role Goals
	**Optimizing child's mental and physical comfort:** Attempts by parent to improve the child's comfort through reassurance and physical actions that require presence	Goals Beliefs about consequences
	**Being involved in hands-on care:** Parental perceptions that they can and should be involved in their child's physical care activities, and efforts to do so	Skills Beliefs about capabilities
Ability to fulfill the parent role in PICU	**Self-identity as important to child's medical care**: Perception or acknowledgement by the parent that their child's medical care is improved by their presence or actions when they are present	Social/professional identity and role Beliefs about consequences Beliefs about capabilities Skills
	**Receiving opportunities to fulfill the parent role**: by the PICU team when parents are present	Reinforcement Social/professional identity and role Social influences
	**Expectations and obligations of a parent**: Statements of what a parent ought to be doing with respect to presence to ensure their child's welfare	Social/professional identity and role Goals Emotion Social influences Intentions
	**Feeling diminished as a parent**: Perceptions of loss of the parent role, with loss of things to do for the child	Social/professional identity and role
Importance of being there for the child	**Just being there for the child**: the perception that just being present with and for the child is an important parent role and contributes to good parenting	Goals Intentions Social/professional identity and role
	**Fear of not being there for the child during an important event:** Parent concerns about not being at the bedside for their child when an important event occurs, either related to progress and improvement or to deterioration	Beliefs about consequences
**Theme: Life Beyond the Hospital** **Description:** Aspects of the parent's life outside of the hospital, including family life, that may impact the abilities, drive, and opportunities at the bedside “So, you know, you feel like you would love to be there, but you're also needed at home.” (Participant 9)		
**Subthemes: Factors identified as barriers or enablers to parental presence**		**TDF Domain**
**Financial challenges:** Financial stresses caused or worsened by parental presence in the hospital with their child		Environmental context and resources Beliefs about consequences
**Distance to travel to the hospital:** Distance from home to the hospital where the child is admitted to PICU		Environmental context and resources
External responsibilities	**Work responsibilities:** The interaction between the parent's employment and presence including expectations from employers, colleagues, and those parents place on themselves.	Environmental context and resources Social/professional identity and role
	**Family responsibilities including childcare:** Responsibilities parents have to other members of the immediate and extended family including parental duty of care for other children and ability parents have to fulfil these responsibilities.	Environmental context and resources Social/professional identity and role Social influences
**Theme: Parental Intrinsic Responses and Coping** **Description:** Emotions stemming from the child's PICU admission, strategies used by the parent to cope with these emotions, and how these impact the time spent at the bedside. “It can feel like you're losing a little bit of your sanity. And then when you step away for a little while, you're like, oh, I feel better now.” (Participant 10)		
**Subthemes: Factors identified as barriers or enablers to parental presence**		**TDF Domain**
**Self-imposed expectations**: The expectations that parents place upon themselves to remain in PICU with their child		Intentions Social/professional identity and role
**Ability to remain in PICU**: Parent's self-identified inherent ability to stay in the PICU		Beliefs about capabilities Social influences
Parental self-care	**The need for a mental health break**: parent-identified need to leave PICU to refresh and enable coping	Beliefs about capabilities Beliefs about consequences Emotions Environmental context and resources Memory and attention
	**Need for distraction**: need for distracting activities to engage the mind either in the PICU or to distract from thinking about PICU	Environmental context and resources Emotion Memory and attention
	**Encouragement from others to engage in self-care**: Support from PICU team members or family and friends to engage in self-care activities, including encouragement to leave the bedside to do this	Social influences
	**Attention to basic needs**: Parent recognition of need for, and strategies taken to secure, basic necessities including sleep, food, and hygiene	Memory and attention Environmental context and resources Goals Intentions
	**Parental intrinsic ability to trust their child care to others**: Ability the individual parent to trust others, medical and otherwise, to care for their child(ren) which allows them to either stay at the bedside (care of other children at home) or to take breaks from the bedside	Belief about capabilities
PICU-triggered emotions	**Empathy for the child's experience**: related to experiences witnessing the child's experience	Emotion
	**Feelings of stress, anxiety, and fear**: feelings that are engendered either by presence at the bedside, or by being separated	Emotion Knowledge
	**Sense of helplessness**: sense of being unable to affect the child's care or make a difference, which may be either a barrier or a facilitator to presence	Beliefs about capabilities Emotion Optimism
	**Sense of guilt and obligation:** related to either being presence or absent	Emotion Intentions
	**Seeking peace of mind:** seeking to feel relief by being at the bedside with the child, able to reassure themselves	Reinforcement
	**Experiencing joy/despair**: as they impact presence at the bedside or as a result of being at or away from the child at the bedside	Emotion Beliefs about capabilities
**Theme: Support Structures** **Description:** Hospital, service-based, or individual supports and support systems that may impact parental capability, opportunities, and motivation to remain at the bedside with their critically ill child “That's what I think of when I arrived here, I asked myself, I hope that my son and [wife] is here. Because, you know, it's not just only a physical one but emotional one also that they can support me, right. Like sometimes I feel losing hope, right. Like oh, my goodness, it's really hard to be alone. And, you know, it's really nice seeing other people.” (Participant 5)		
**Subthemes: Factors identified as barriers or enablers to parental presence**		**TDF Domain**
Support from PICU team	**PICU support for logistics and basic necessities**: Assistance from any PICU team member or through the hospital to support access to sleep, food, hygiene, and to enable parent self-care in the room with their child	Social influences Environmental context and resources
	**Emotionally supportive PICU staff**: Staff attention to and support of parents’ emotional needs in PICU	Social influences
Support of family and friends	**Family support at the bedside**: Support provided by other family members at the bedside and the importance of that support	Social influences
	**Family and friends who bring items to make it possible to stay**: Attention to parent's needs through provisions by individuals outside the hospital	Social influences
	**Help with external responsibilities**: Assistance to ensure that family life, including care of siblings, continues while the child is in PICU	Social influences
	**Emotional support outside hospital walls**: Emotional and mental health support from those who are not at the bedside	Social influences
**Theme: The PICU Environment** **Description:** the impact that both the physical and non-physical environment of the hospital and PICU have on parental capability, opportunities, and motivation to remain at the bedside with their critically ill child. “I think it's the facility. I think the way it's set up is amazing. It's really warm, and it's… You know, it's not as clinical. It's inviting. And even to have the chairs at the back… They have the bed positioned so that the nurse can see through the glass. And then they have the area at the back for the families.” (Participant 4) They allow me to spend as much time as I want to be there with him. The only thing they would ask is if I would like to step out for an x-ray for my own health. I don't have to, but I usually do. But other than that, no, they're very open and you could basically be there 24/7, 365 if you wanted. (Participant 9)		
**Subthemes: Factors identified as barriers or enablers to parental presence**		**TDF Domain**
**Hospital policies and practices**: Rules and regulations from the hospital and PICU about parental presence and the manner in which they are applied by the PICU team		Environmental context and resources Social influences
**Witnessing other children and families:** Experience of witnessing the critical care stays and events for other families and patients		Environmental context and resources Emotion
**Belief that presence impacts the work of staff:** Parental perceptions that their presence may impact PICU clinicians and their work		Beliefs about consequences
**Familiarity with PICU team and processes**: Experience that parents may have already had in the PICU and how familiar they are with the PICU space, context, processes, and team		Knowledge
**Perceived trustworthiness of PICU team**: How the parents perceives the medical knowledge, skills, and abilities of the medical team to care for their child		Beliefs about capabilities Beliefs about consequences
Staff attitudes and behaviors	**Perceived expectations and judgment**: Related to the degree and type of the individual parent's presence and involvement	Social influences
	**Staff approach to parental presence and engagement**: The general approach to parental presence, either positive or negative, taken by staff and the impact of general staff attitudes and behaviors on parental willingness or ability to remain at the bedside	Social influences Environmental context and resources
The built environment	**Potentially unfamiliar medical equipment and technology**: Technology within the PICU space, including sensory experiences with the technology, exclusive of medical equipment directly in or on their child or the interaction between the child and equipment	Knowledge Environmental context and resources
	**Ease of PICU access within the hospital**: Ease of gaining access to the PICU within the hospital complex	Environmental context and resources
	**Noise:** Sounds within the PICU and hospital environment and their impact on parental presence	Environmental context and resources
	**Comfortable and functional physical environment**: Furniture, appliances, design and other physical aspects of PICU rooms and parent spaces that affect presence	Environmental context and resources
	**Privacy**: Parents’ expressed need for privacy and perception of how the physical space of the PICU affects their child and family's privacy	Environmental context and resources

### 3.1 Understanding the medicalized child

Some participants noted that it was more difficult to spend time with their child when they perceived them to be “very ill” including when the child was attached to lines/wires and equipment, or when their child was upset or in pain. An exception to this was one father who felt that his child might die, and subsequently was motivated to be present by the increasing severity of his son's condition: “Like if this is the last time I get to see my son, I want him to know I was at least there. And I don't want to lose any more time with him than I could.” (Participant 7). Parents with more knowledge of their child's critical illness and the PICU expressed greater ease with being at the bedside, which enabled presence. Parents wanted information about their child's condition and recognized that presence resulted in opportunities for information and improved understanding about their child's clinical condition. “Well, it's just like if you come and if you don't know all the answers, like you don't know how it's going to turn out, it can be petrifying. So it's great that they explain everything and keep you involved.” (Participant 1).

### 3.2 Maintaining the parent role

Although parents in the PICU identified significant changes in the tasks they could do for their child, their identity as a parent was largely unchanged and parents strove to maintain their parent role through presence with their critically ill child. One father stated, “I'm just doing what I'm supposed to be doing as a father—taking care of my child.” (Participant 9). Many parents similarly expressed feeling a sense of responsibility to their child. They stated that it was their duty to spend time at the bedside, even if that meant “just being there” without an active role. Some parents perceived parental role limitations in the PICU. This was seen primarily as a barrier to spending time with their child. One mother said, “Right now it's all of them doing it. Like I'm just… I feel like I'm just decoration in the room.” (Participant 10). However, parents were motivated, and therefore enabled, to be present to facilitate engagement through advocacy, providing comfort, and hands-on participation in care. Parents found it helpful when staff let them know ways to stay involved. One father said, “I would rather them do it anyways because they're more trained. But like yesterday…one of the nurses… was redoing his bandages. And she was asking me to help. And that made me feel good….Because I felt like I was doing something to help my son” (Participant 7). The primary driver of presence for multiple respondents was a sense that their child needed them emotionally and a perception that they supported their child's comfort, particularly during changes or significant events. “For me, I want to be close with him because that way if he needs anything or he wants comfort, I'm there.” (Participant 9).

### 3.3 Life beyond the hospital

Outside responsibilities, primarily family and work, were barriers to presence and continued largely unabated during a PICU admission. Parents expressed varying abilities and willingness to disconnect from them. “He was supposed to go back to work yesterday. So we've kind of been thinking about that, and calling people and trying to figure all that out for him to be off so he can stay with us here.” (Participant 8). Multiple participants discussed the financial difficulties imposed by a hospital admission and the need for the parent to be at the bedside. One parent summed it up as: “It is tough because when your child gets sick, your bills still keep going.” (Participant 6M).

### 3.4 Parental intrinsic responses and coping

For most participants, the child's illness triggered stress and anxiety-based responses that impacted their willingness and ability to remain at the bedside. Multiple parents expressed that presence with their child was a method of coping with the fear and stress that their child's illness brought. For others, being in the PICU caused anxiety that made it difficult to remain, though absence resulted in feelings of guilt. “I feel like because it's so stressful to be in there with him, it's like when we do spend time away, we kind of get…like feel guilty because we're not spending all of our time with him. But sometimes it's just it's really hard because like with all that stuff going on, it's very overwhelming.” (Participant 3M).

The primary coping mechanism described by participants was taking time to engage in self-care. The need for self-care was identified in factors across multiple domains including Beliefs about Capabilities, Social Influences, and Memory, Attention and Decision Processes. Self-care included basic functions like eating, sleeping, hygiene, and time away to focus on mental well-being. As explained by one mother: “it can feel like you're losing a little bit of your sanity. And then when you step away for a little while, you're like, oh, I feel better now.” (Participant 10). Self-care was facilitated by encouragement from staff and other family members, and was impacted by the parent's self-assessed ability to trust others with their child. One parent stated, “I'm not very good at taking breaks at all. I'm a social worker, and I encourage families in my own practice to self-care. And I'm not good at it myself. It's just hard asking for help and saying that you need help.” (Participant 4). While this ultimately resulted in time spent away from their children, parents noted that breaks from the bedside/unit helped them feel like they were better equipped to care for their children, resulting in facilitated and engaged presence: “But if he doesn't get any sleep or if he doesn't eat, we're not going to be any good to [son].” (Participant 6M).

### 3.5 Support structures

Support structures helped parents spend quality time with their children and ensured the child always had a loving family member present. These structures also facilitated self-care as demonstrated by one mother, who was at bedside with her husband: “So it's the support that we can kind of lean off each other… Like I can go down and make the lunch or coffee, and he can sit by the bed, or vice versa, whatever….You know, I've been a single mother so I know how tough it is. So it really, really helps that we're together… Because, you know, there are some places that you can only have one support person [for the child].” (Participant 6M). Parents identified emotional support from family and PICU staff as important for maintaining wellness during a PICU admission. All participants discussed real or potential detrimental impacts of restricting family presence to one individual. One mother who was apart from her partner (not the child's other parent) because of COVID-19-related restrictions stated: “I mean it would be nice if my boyfriend could be here, or my mom. And I understand that they can't. I get that. But that's the biggest thing right now that's making it the most challenging, is that I'm here alone.” (Participant 10). Support structures helped with accessing basic supplies and taking care of family, work, and financial responsibilities, which allowed parents to remain with their children.

### 3.6 The PICU environment

The PICU environment was conceptualized as both the physical and intangible environment. This included overt and latent messaging parents receive about their presence from rules and policies, unit practices, and staff cultures, attitudes, and behaviors. The physical space included noxious experiences like beeping, alarms, and the appearance of unfamiliar medical technology that made presence very difficult. One mother stated, “And to see the screens all the time, watching them constantly, that's hard. Because you're aware of the numbers and what they should be at. And every beep or every sound, you panic.” (Participant 10). On the other hand, almost all parents discussed how a comfortable and functional physical environment supported their presence with their child by providing necessities and avenues for self-care throughout their child's admission. As one parent noted: “And I guess things that also would be helpful are the furniture is helpful. You know, they have comfort [sic] places for you to sit. And they offer pillows and warm blankets and things to make you more comfortable while you're staying all day and watching and waiting.” (Participant 11).

The attitudes and behaviors of the PICU team members were highly impactful on the opportunities and motivations of parents to remain at their child's bedside. Parents who felt judged were more likely to remain at the bedside but did not partake as willingly in self-care activities that enabled their engagement. As stated by one mother: “Because I feel like if I leave, I'm being perceived as not caring.” (Participant 4). Parents who felt embraced and welcomed to participate also perceived that it increased their presence, and increased their trust in the medical team so that they could leave if needed. One mother felt she could leave because the staff called when they said they would: “And the staff did call me if, you know, I was missing something. And I left instructions to say, you know, please call me if something changes. So I felt okay to then leave for a few minutes or to go sleep in that room and come back.” (Participant 11).

## 4 Discussion

Using a theoretical domains framework to inform our qualitative descriptive inquiry, we identified that parents’ ability, motivation, and opportunities to be at the bedside of their critically ill child in the PICU are impacted by factors that are both intrinsic and extrinsic to themselves. These factors were captured by six themes: *Understanding the Medicalized Child*; *Maintaining the Parent Role; Life Beyond the Hospital; Parental Intrinsic Responses and Coping; Support Structures;* and *The PICU Environment*. This study provides important insight into the emotions, coping strategies, and social structures that parents perceive impact their presence at the bedside.

Dudley and Carr examined the experience of parents who were vigilant at the bedside of their hospitalized child in a general pediatric setting (24). Vigilant parents were motivated to remain present by a commitment to their child's care (*Understanding the Medicalized Child)* and required self-care to persevere and maintain resilience (*Parent's Intrinsic Response and Coping*). They also experienced emotional upheaval (*Parental Intrinsic Response and Coping)*, evolving relationships with family and healthcare staff (*Life Beyond the Hospital, The PICU Environment)*, and changes in their environment and daily life (*Life Beyond the Hospital)*. Several of our findings map onto these themes, though are context-specific for the PICU. Additionally, we identified the importance of support structures and emphasized how maintaining the parent role can enable presence. Using a directed content approach, we were able to specify individual sub-themes that function as barriers or enablers to presence and that may be amenable to behavioral change interventions.

While our group has previously examined the correlation between factors external to the parent and amount of time spent at the bedside (14), we sought improved understanding of psychosocial drivers for parents to remain at their child's PICU bedside. Consistent with a growing body of literature (1, 25), all parents identified the importance of maintaining their parent role and identified its loss as a barrier to spending quality time with their children. “Good-parent” beliefs are a parent's beliefs about what is needed to fulfill their own, internal definition of being a good parent to their child (26). Most studies seeking to conceptualize the construct have included a version of “need to remain at my child's side” (27–29). Feudtner proposed that core “good-parent” beliefs in a PICU context include ensuring the child feels loved, attention to the child's health, advocacy and being informed, and spiritual well-being (30). Our participants subscribed to three of these four beliefs as enabling their presence. We propose that the drive to be a “good parent” enables and promotes bedside presence in the PICU, which then supports parental self-identity as a good parent. This is, not least, through the opportunities that presence provides for comforting the child as well as for direct engagement in the child's care.

We identified several specific emotional responses that influenced parental presence. Stress and anxiety are consistent features of the parental experience in PICU (31, 32) and were potential barriers for most participants. Most participants also described despair and pain in empathizing with their child's experience but maintained presence because of the primacy of fulfilling the parent role. Where the need to alleviate stress overtook the parent role, parents experienced guilt, consistent with good-parent beliefs of “putting my child's needs above my own” (28), and which pulled them back to the bedside. As demonstrated by Participant 3M above (Parental Intrinsic Responses and Coping), participants in this study similarly expressed similar feelings of guilt.

The importance of self-care activities as a form of coping was a transversal theme. A recent qualitative study by Jarvis and colleagues explored ways of supporting family members in the PICU and noted that family members should be intentional about performing self-care activities (33). Although self-care activities may remove parents from the bedside, they enable active and engaged presence. In keeping with findings from Ames and colleagues, engagement optimizes parents’ ability to maintain their parent role in the PICU (9), which may have positive longer term emotional impacts. Engagement allows realization of a truly family-centred model, where the parent participates as a core member of the child's team rather than as a passive observer (34). Policies, practices, unit cultures, and individual attitudes that allow the parent to be more fully engaged in decision-making, information-gathering, and hands-on care of the child may facilitate presence. When parents are engaged they may have improved understanding of their medicalized child, maintenance of the parent role in PICU, and also decreasing parental stress (35). Hospitals and health care professionals can support parents’ need for self-care activities by having comfortable, family-centred facilities (36) in close proximity to the child, allowing freedom of movement within the hospital complex, and accessible food options. Reminders to parents of the importance of taking a break may not adequately facilitate self-care when a parent does not trust their child's care to others. Thus, PICUs must focus on building trustworthiness and consistently approaching parents and families with non-judgmental compassion.

The TDF was a useful scaffolding for soliciting parents’ perceptions of barriers and enablers to their presence and allowing a fulsome understanding of areas in which parents may require support and also where intrinsic strengths allow the parent to cope with the trauma of a PICU admission. The significance of parental presence in PICU extends beyond the child's PICU admission itself. As conceptualized by the Post Intensive Care Syndrome-Pediatric (PICS-p) framework, a PICU admission may have long-lasting negative health sequelae for both the child and their family members (37). The profound impact of the child's critical illness and the experience of PICU care may result in significant negative emotional health sequelae for parents both present and not (38). In turn, parental presence and responses to the admission may impact the health of their child (39). Optimally supported presence has the potential to mitigate some of the PICS-p morbidities. Our thematic findings compliment those from Jarvis's study to identify ways of optimally supporting families of critically ill children in PICU (33), but include additional details about factors influencing presence.

This study is strengthened by its methodologic rigor, as well as parent partner involvement in the development of study materials, protocol, and data analysis. Although we sought maximum variation in our sampling strategy, the primary limitation of this study is that we were unable to sample parents who were not present in the PICU who may have experienced unexplored barriers to their presence. Additionally, our interviews were limited to English speakers. It is likely that a language barrier negatively influences the experience of bedside presence including interactions with and trust in staff, access to culturally appropriate food and sleeping arrangements, and involvement in the child's care. We did not collect information on economic security, race, or ethnicity. These may impact the barriers or enablers to presence for individuals. In particular, one might predict that *Life Beyond the Hospital*, parents’ ability to trust their child's care to others, and themes related to staff attitudes and behaviors may take on different significance with a demographically diverse sample, and should be explored in future work. Our results may also be context-specific. This research was conducted at a single centre during the COVID-19 pandemic. Although all participants were allowed to have one support person, restrictions to sibling presence may have forced some parents apart. The pandemic itself altered work and social stresses that may have impacted the results, such as the emphasis on social supports.

Future work: Using the relevant TDF domains, future work may leverage these findings to design domain-based interventions aimed at optimizing parental presence in the PICU. This may include such interventions as routine PICU orientation processes, resources to explain and demystify PICU terminology and technology, and a screening tool to identify person-centered potential barriers to engaged parental presence. Future work to optimize family presence should include an assessment of the impacts of interventions on presence, engaged presence, and health outcomes, notably PICS-p for children and their parents.

### 4.1 Parent partner response

As parents to a young child with several weeks-long PICU admissions, planned and unplanned, it is interesting how this study highlights familiar experiences and wonderful that it points to ways to make PICU admissions easier for families. Our PICU stays were pervaded by feelings of helplessness: an inability to comfort our suffering child, not understanding the medical equipment or treatments being offered, and a constant fear that our child would die despite everyone's best efforts. Feelings of helplessness were compounded by other pressures: Demands of work and family, desire to make things as “normal” as possible for your own child, and the discomfort of being “on display”, surrounded by health care providers nearly 24-7. We also questioned ourselves: Were we doing the right things? Did we spend too much time at bedside? Too much time away? Was it wrong to not want to witness a procedure? Were we in the way if we stayed for them? Did family and friends who visited our child hinder delivery of care? We also realized the importance of self-care: comfortable places to sleep and shower, laundry facilities, access to nourishing food—sometimes at odd hours—and a strong internet connection that allowed us to communicate with family, keep up with work, or just try to relax with a movie.

Respondents’ feedback along with our own experiences also point to ways that PICU policies might better alleviate the stress of parents/caregivers. Thinking of our child's earliest admissions, we believe there is space in PICU policy for more deliberate communications—for example, descriptions early on of what the medical equipment does, and what the various beeps and indicators mean (maybe even simple descriptions/diagrams on the wall explaining commonly-used technology and their noises?). Additionally, we've often thought that the PICU might have better prepared us for life after discharge. One aspect of our PICU experience for which we were entirely unprepared was the trauma that followed us home. After one particularly stressful admission, a physician commented that “all's well that ends well.” While this was true in the moment, all was not well as our PICU experience continued to haunt us in the form of our child's ongoing nightmares, or when an innocuous and unexpected beep evokes PICU machinery, transporting us back to that terrible time, or in the profound dread that makes every new illness a reminder of how easily it could all happen again. Better preparing parents for the long-term impact of a PICU stay would be a very welcomed support.

## 5 Conclusions

Parental presence at the bedside in the PICU is facilitated or hindered by factors related to Understanding the Medicalized Child, Maintaining the Parent Role, Life Beyond the Hospital, Intrinsic Responses and Coping, Support Structures, and The PICU Environment. This work identifies factors impacting presence that are amenable to change and quality improvement. The TDF should be leveraged to design initiatives that support parents and facilitate engaged parental presence in PICU, which may decrease adverse long term health sequelae in both parents and critically ill children.

## Data Availability

The datasets for this article are not publicly available due to concerns regarding participant/patient anonymity. Requests to access the datasets should be directed to the corresponding author.
