# Supplementary material for: “I want to be there. I have to be there.”: Parents’ perceived barriers and facilitators to bedside presence in the pediatric intensive care unit
Source: Front Pediatr. 2024 Jan 8;11:1308682. doi: 10.3389/fped.2023.1308682 (PMC10800939; doi:10.3389/fped.2023.1308682)
Supplement: Supplementary file 2 [file Datasheet2.docx]

**Supplemental File 2: Study team reflexivity**

EP, JF, MR, and JC are cis-gendered white females who live and work on unceded Mi’kmaw territory in the Atlantic region of Canada. EP is a medical doctor who was a medical student at the time of the study and is now a specialty trainee in pediatrics. JF is a mid-career PICU physician with both qualitative and quantitative research experience and significant research and clinical supervisory experience. MR is an early career research coordinator who has a master’s degree in public health and significant qualitative experience. JC is a senior PhD researcher who has qualitative and mixed-methods expertise including use of the TDF. JAS is a mid-career child health epidemiologist and biostatistician, who has a PhD in sociology and conducts research in qualitative methodology. CS and MW are parents of a previous PICU patient and take a keen interest in hospital policy.
