# Supplementary material for: “I want to be there. I have to be there.”: Parents’ perceived barriers and facilitators to bedside presence in the pediatric intensive care unit
Source: Front Pediatr. 2024 Jan 8;11:1308682. doi: 10.3389/fped.2023.1308682 (PMC10800939; doi:10.3389/fped.2023.1308682)
Supplement: Supplementary file 3 [file Datasheet3.docx]

Supplemental File 3: Final Interview Guide

**Parent/Caregiver Interviews**

**Interview Guide**

Explanation

Thank you for agreeing to speak with me today. This interview should take approximately 30 to 60 minutes and will be audio taped to ensure that I accurately capture all of the key points that you share with me. Any identifying information (for example your name, or your child’s name) that you use in the course of our discussion will be removed from the interview transcripts. There are no right or wrong answers to these questions. We are interested in understanding the things that made it easy or hard for you to visit and spend time with your child while your child has been sick in the intensive care unit and how you’ve felt about visiting and spending time in your child’s room. If you wish to end the interview before I have asked all of the questions or if you wish to withdraw from the study at any time during the interview you are free to do so.

Now that you have been through an ICU admission with your child, you are an expert in it. We know that different families have different desires and abilities to stay with their children in the PICU, so we are looking for your thoughts on things that may make it easy or difficult, important or less important for families to stay with their child in the PICU, and what you think about how this impacts children at different points in their illness.

Note to interviewer: Ask the primary numbered question. Follow the flow. Lettered questions are possible follow-up questions. When parents discuss other aspects of the general PICU stay, ask “how do you think this impacted your presence with your child in the PICU?”

***Italicized questions were added because of concurrent analysis*

1. Can you tell me a little bit about how your child came to the PICU?
   1. How long ago?
   2. How sick were they?
   3. How long before your child’s PICU admission did you know that he/she would be admitted to the PICU?
   4. Did you come with your child to the PICU when they were admitted? Or did you come in later?

**Pause to re-focus interview on PARENTAL PRESENCE**

1. Can you tell me a little bit about what it is like being present with your child in the PICU?
   1. What made it easy to be there?
   2. What made it difficult to be there?
   3. What was your sleep like while your child was in the PICU?
   4. What were your meals like while your child was in the PICU?
2. How have COVID-19 restrictions impacted your ability to spend time with your child in the PICU?
   1. *Are there other family members who would have been present if it weren’t for COVID-19?*
   2. *How have COVID-19 restrictions impacted your ability to focus on your child during their PICU stay?*
3. Was this the first time your child has been admitted to the PICU?
   1. Have you ever had another child admitted to the PICU?

For parents who have been to PICU before:

1. How was staying with your child similar between the two admissions?
2. How was staying with your child different between the two admissions?

b. How do you think being with your child might change if you had to do it again?

1. *Did you feel prepared to be at the bedside with your child in PICU?*
   1. *If yes, who/what helped to prepare you?*
   2. *If no, what could have been done to make you feel more prepared?*
2. Do you think that there are special skills or knowledge that parents need to have to be at the bedside of their child?
3. How do you think a parent’s abilities affect the time they spend with their child in PICU?
4. How did you see your role for or with your child in the PICU?
   1. *How has your role changed being in the PICU?*
      1. *How do you think being in the PICU with your child, or being out of the PICU impacts your ability to stay updated?*
      2. *How does your presence impact your participation in medical decisions?*
5. When your child was first admitted/when you were preparing for your child’s admission, what were your intentions for spending time with him/her?
6. Can you tell me about your goals around staying in the PICU with your child?
   1. *What kind of things make it easier/harder for you to participate in your child’s care?*
7. What do you think are the impacts of a parent spending time with their child in the PICU? On your child? On you? On staff?
8. How do PICU staff influence the amount and quality of time you spend with your child in PICU?
   1. *How do their behaviours influence it?*
   2. *How does trust influence the amount and quality of time you spend at your child’s bedside?*
9. *Do you feel like there are certain things that you need so that you can be at your best when you are present with your child in PICU?*
10. *Did you feel like other people had expectations about how much time you would spend with your child?*

*If yes:*

- 1. *How did these expectations influence the amount/quality of time that you spent with your child?*

1. *How does your child’s medical status impact your ability to be present at the bedside?*
2. *How did you feel when you weren’t at your child’s bedside?*
3. Can you tell me about a time that you visited with your child that was easy? (repeat questions for a time that was difficult)
   1. What made it easy?
   2. What was happening with you that might have made it easy?
   3. Was there anything in particular happening with your child that might have made it easy?
   4. Was there anything about the PICU environment that day that made it easy?
4. *What are the personal qualities that you have that makes it easier or harder to be at your child’s bedside?*
5. On a scale of 1-10 with 1 being not at all important and 10 being very important…how important do you think it is for parents to be present at the bedside while their child is in PICU? Can you tell me why you think this?
