# Supplementary material for: “I want to be there. I have to be there.”: Parents’ perceived barriers and facilitators to bedside presence in the pediatric intensive care unit
Source: Front Pediatr. 2024 Jan 8;11:1308682. doi: 10.3389/fped.2023.1308682 (PMC10800939; doi:10.3389/fped.2023.1308682)
Supplement: Supplementary file 4 [file Datasheet4.docx]

Supplemental File 4: Themes and subthemes with representative quotations

| **Theme: The Medicalized Child** | |
| --- | --- |
| *Subthemes* | *Representative quotation* |
| Information needs | |
| Medical or PICU experience and knowledge | I mean if I wasn't here... I mean I know my child better than anybody. I don't know what's all going on with all of his injuries, of course, but I know what he likes and what he doesn't like. I know what he could do beforehand. Where they never met him before  Participant 10 |
| Preparation for appearance of critically ill child in PICU | R1: I mean they could have pre-warned us a little bit about all like the tubing and stuff, and all the wires that were going to be connected to him.  R2: Maybe like take one of those robot baby kind of things and hook up all the wires and stuff so we can see what it's going to look like, kind of thing. So it's hands-on, like this is what your son's going to look like when we go in the PICU.  Participant 3M and 3F |
| Opportunities to gain information | So probably in a day I tried to visit him five times, but I stay for just only like 15 minutes to get all the information. Like oh, what's the update? And after I got all the updates, I want to go back at my room and do something instead.  Participant 5 |
| Knowledge of the child's baseline and disease process | So sometimes we let them know when we've seen something out of the ordinary. Because sometimes it might not be normal for [daughter].  Participant 4 |
| Child’s clinical course | |
| Preparation for child's clinical course and outcome | And I guess because we knew what she was coming for. We knew what the outcome was going to be. I mean maybe, yes, it could be different if it was an emergency situation or if our child was really sick and we didn't know what was going on with the child. But we're lucky in the case that we didn't know what was going on. We knew what had to be done, and we knew what the outcome was going to be.  Participant 2M |
| Child's medical status | I don't know how to explain, but it's really hard if I'm seeing him, you know, with kind of tube and apparatus. But I said to my wife, probably I can stay longer if those apparatus or the tube is already removed.  Participant 5 |
| Hope for recovery | Yeah, it's a lot easier when she’s doing well. Like she's doing a lot better now. And a lot of the machines are taken away and taken off of her. So it's way easier to be in there and see her kind of looking like her old self a little bit than it was to see her hooked up to all those things and sedated and that kind of thing. So it's definitely easier to be in there when she's doing better and looking better and feeling better.  Participant 8 |
| Witnessing the child’s response to PICU | |
| Child's responses to care | Or you walk in, you see him relaxed. You know, he looks extremely comfortable. That's a lot easier to walk in there…  Participant 6F |
| Child's expectations | Because [son], I know he assumes that mom and dad’s going to be there. That’s a given.  Participant 9 |
| **Theme: PICU Parent Role** | |
| *Subthemes* | *Representative quotation* |
| Perceived roles and responsibilities while their child is in PICU | |
| Advocating for the child | Like being right there and being there when they come around to do rounds and that kind of thing has been good because we've been able to catch them and kind of let them know if we have any ideas or suggestions or anything that we've seen.  Participant 8 |
| Optimizing child's mental and physical comfort | Because they need that support. Like it's not just us going through it, like it's him going through it. And knowing that like even just us being there, we can do something, even try to remotely comfort, is very important. And it keeps us in the loop as well.  Participant 3M |
| Being involved in hands-on care | I mean I know how to, you know, reconnect the blood pressure cuff or fix one of his cardiac monitors. I can do those kind of things. Like if the stickers pop off or his O2 stat comes off, I can do those kind of things. But I don’t really need special skills.  Participant 10 |
| Ability to fulfill the parent role | |
| Self-identity as important to child's medical care | And I guess our role, I guess, we’re still parents, still asking questions, making sure, you know, she was getting what she needed to get.  Participant 2F  I can't do anything. Like they'll do everything. They change her, they bathe her, they do her meds. I'm not in control at all  Participant 4 |
| Receiving opportunities to fulfill the parent role | But then also sometimes as he gets better, so now I can hold him and things. So then I want to be here for whenever he's awake and needs to do that.  Participant 11 |
| Expectations and obligations of a parent | But I was there willingly to help, to do anything that I could because it's my child.  Participant 2M  Well, when you’re a father or a mother that is your responsibility. You take care of your children. Children come first. Everything else is second.  Participant 9 |
| Feeling diminished as a parent | I think they would be, “What are you doing sitting there for 12 hours a day?” You can only pace around the floor so many times, and fill out the bravery bead sheet. Fluff a blanket, I guess.  Participant 10 |
| Importance of just being there for the child | |
| Just being there for the child | My role is her mom and her caregiver and her number one support, the whole time. That’s my role.  Participant 1 |
| Fear of not being there for the child during an important event | Like yesterday I didn't feel good leaving him at all because I didn't know… I knew that if something happened and they had to extubate him early, they just would. But I wanted to be there for it just in case something happened during the extubation.  Participant 10 |
| **Theme: Life Beyond the Hospital** | |
| *Subthemes* | *Representative quotation* |
| Financial challenges | So it's very difficult financially because if I don't work, I don't get paid. So it is very difficult. Because I get three sick days a year.  Participant 6M |
| Distance to travel to the hospital | Like we're lucky that we got to stay up here. Because if not, then we live about 45 to 50 minutes away, so that is a quite lengthy drive. So being actually like right across the hallway, we're able to spend more time with him than if we were to be home, if we weren't able to be here.  Participant 3M |
| External responsibilities | |
| Work responsibilities | Even for me not to be working right now, I feel guilty. I should have gone back to work today. And I feel guilty because I feel like I'm having people at work cover for me when I'm here looking after my daughter.  Participant 4 |
| Family responsibilities including childcare | We alternate back and forth. So he has mom here, and I’m at home with the kids. Then vice versa. That way both set of kids right now… You know what I mean? That the child that’s at the IWK has mom and dad both, and the children at home have mom and dad both to keep it as stable as possible.  Participant 9 |
| **Theme: Parental intrinsic responses and coping** | |
| *Subthemes* | *Representative quotation* |
| Self-imposed expectations | I would say you were thinking that you were going to be here full-time, pretty much. R2: Yeah. R1: And I figured that I would be there most time, but in and out.  Participants 2F and 2M |
| Ability to remain in PICU | I'm not very good at taking breaks at all….I encourage families in my own practice to self care. And I'm not good at it myself. It's just hard asking for help and saying that you need help.  Participant 4 |
| Parental self-care | |
| The need for a mental health break | Yeah, because I wasn't like looking right at her and seeing her there. Like if I took a minute and stepped outside and just kind of pulled myself together, it was a little bit easier than staying in there. And I did that a few times.  Participant 8 |
| Need for distraction | I know it sounds little and it's dumb, but when you're alone and you're sitting up here for hours and hours and hours, and you can't have any company, it would be nice if you could throw a TV on, you know.  Participant 1 |
| Encouragement from others to engage in self-care | But the nurses and the RT and the doctor all came in and they literally talked us into it. They’re like, “This is almost like respite. Like we know what we're doing. We'll watch her. We’ll be with her every second. You need a break. Like go have a break.”  Participant 1 |
| Attention to basic needs | But if he doesn't get any sleep or if he doesn't eat, we're not going to be any good to [son].  Participant 6M |
| Parental intrinsic ability to trust their child care to others – medical and otherwise | It's so hard for me just to let somebody else do her care or even give her medications, do her feed.  Participant 4 |
| PICU-triggered emotions | |
| Empathy for the child's experience | And it’s so hard looking at him and seeing him the way he is, and wishing I could be almost in his shoes instead of him. Be there and take his pain away or something I could do for him.  Participant 7 |
| Feelings of stress, anxiety, and fear | It makes us really worried because when he gets stressed out, everything starts beeping because everything starts rising. Like his blood pressure, his heart rate and his breathing and stuff like that. So it's definitely very stressful even just to be in there with them.  Participant 3M |
| Sense of helplessness | I get really emotional, and I cry for no reason. I just cry. And I don't know, I just feel, I don't know, helpless. I can't do anything for her, I can't help her.  Participant 4 |
| Sense of guilt and obligation | So I would feel guilty if I wasn't here. He's my whole world.  Participant 7 |
| Seeking peace of mind | But it was always like in the back of my mind, like I wonder how she's doing. Is she sleeping, how she’s feeling? Like I was always thinking about it when I wasn't in the room with her.  Participant 8 |
| Experiencing joy/despair | I've definitely had more bad days here than good. Like I don't walk in the room and feel positive. Like there’s nothing about the situation that’s happy. It's basically like someone from ripped your heart out.  Participant 7 |
| **Theme: Support Structures** | |
| *Subthemes* | *Representative quotation* |
| Support from PICU team | |
| PICU support for logistics and basic necessities | They have a social worker just for PICU that will come and like get you a room. They’re always good about like finding you a room. They always ask if you need anything. Like they're just super considerate. They are always trying to make it easier for the parents, I think.  Participant 1 |
| Emotionally supportive PICU staff | It's been nice that some of the nurses have reassured me when I've been, you know, sad or afraid. They've explained things.  Participant 4 |
| Support of family and friends | |
| Family support at the bedside | And yeah, just having someone to talk to. Like I'm glad [husband] is here. Like so that we can kind of talk to each other. And I feel like it would be way harder if I was here by myself or something like that.  Participant 8 |
| Family and friends who bring items to make it possible to stay | So to ask him to bring…to continuously bring us supplies and food, that's difficult. So I felt to ask him to do that, it’s hard.  Participant 4 |
| Help with external responsibilities | Yeah, my parents have been… They've been coming up and going to our house and taking care of all the animals and doing that thing. And we also have like a friend who lives next door that's been doing that when my parents can't. And we have people who bring us clothes and that kind of stuff because neither of us really want to leave. We want to be here and stay here. So we have had quite a bit of support from people who bring us what we need, and kind of help us out that way, definitely.  Participant 8 |
| Emotional support outside hospital walls | Like the day of the surgery, we had my mom, her mom, my nan, her cousin. We had quite a few people here just up on the roof kind of just supporting us. So that kind of helps.  Participant 3F |
| **Theme: The PICU Environment** | |
| *Subthemes* | *Representative quotation* |
| Hospital policies and practices | They allow me to spend as much time as I want to be there with him. The only thing they would ask is if I would like to step out for an x-ray for my own health. I don't have to, but I usually do. But other than that, no, they're very open and you could basically be there 24/7, 365 if you wanted.  Participant 9 |
| Witnessing other children and families | …it was fairly quiet for a while. And so we kind of just felt in our own little world. And then when it got busier and you could hear some of the other kids, yeah, it kind of changed the vibe a little bit.  Participant 11 |
| Belief that presence impacts the work of staff | My opinion I think they like having us there because it gives them the freedom to go do their own paperwork and they can do other things.  Participant 6F  Stressful, because like you see all the doctors working and working, working, and you want to help but you know if you try to help, you’re just going to get in the way.  Participant 3D |
| Familiarity with PICU team and processes | So those first few months when she was an infant were probably some of the scariest, most horrible moments in the PICU for us because we weren't familiar, we didn't know. Like it was our first time in PICU. So now, like eight years later, it's…I mean I feel like I work here.  Participant 1 |
| Perceived trustworthiness of PICU team | But I feel like if there was any place that you could leave your child and have to go, then this would be the place for sure. A hundred percent, I would trust everybody here.  Participant 1  So I mean you can't advocate for your child if you're not here to know what's going on. You just can't. You know, there's sometimes when… The doctors just aren't always right. They’re not. They’re people. They're human. And so a mother's instinct is usually right on, for the most part. It hasn’t failed me yet.  Participant 10 |
| Staff attitudes and behaviors | |
| Perceived expectations and judgment | Because I feel like if I leave, I'm being perceived as not caring.  Participant 4 |
| Staff approach to parental presence and engagement | They ask me afterwards if I have any questions or if I think something or have any ideas. They involve me. And there are some staff that you ask a question to or voice your opinion, and they’re kind of condescending. And I've experienced it in the PICU.  Participant 4 |
| The build environment | |
| Potentially unfamiliar medical equipment and technology | What kind of things do you think you experienced previously that are helping you now? R: Just like the machinery. Your IV towers and the ventilator and all the lines, and the beeping, and the monitors, and the medical terminology - all of that. His father is completely overwhelmed because this is his first experience like with his child, of course.  Participant 10 |
| Ease of PICU access within the hospital | We've got access cards that gets us in through doors from one of the main lobbies downstairs... And, you know, being able to move freely and still obviously be within COVID protocols and all that stuff is excellent.  Participant 2F  [Social worker] had said, “Yeah, like the sleep rooms are a must. They need to have more of those.” Because it does give you… Like you’re close. And so that you could be there in like two seconds or less, you know. So that made me feel able to leave for a few minutes at a time, and able to get some sleep.  Participant 11 |
| Noise | Like sometimes all the alarms are going off, and the nurses will come in – a lot of them. And it starts to worry you. And it's overwhelming sometimes.  Participant 7 |
| Comfortable and functional physical environment | The ICU room itself, you know, the rocking chair so you can actually…or recliner so you can actually sit with her. The couch in the back so you can actually relax. And, you know, you can get up, come and go, kind of thing, up and down from her as needed or as you want. And having full access.  Participant 2F |
| Privacy | So we do hang out at the back more. And it's very private. They do their thing, and we are able to sit there and just chill, just to be in the room.  Participant 4 |

Abbreviations: PICU = Pediatric Intensive Care Unit
